# Neural Stem Cells (NSCs) and Proteomics*

**DOI:** 10.1074/mcp.O115.052704

**Published:** 2015-10-26

**Authors:** Lorelei D. Shoemaker, Harley I. Kornblum

**Affiliations:** From the ‡Department of Neurosurgery, Stanford Neuromolecular Innovation Program, Stanford University, 300 Pasteur Drive, Stanford, CA 94305;; §NPI-Semel Institute for Neuroscience & Human Behavior, Departments of Psychiatry and Biobehavioral Sciences, and of Molecular and Medical Pharmacology, The Molecular Biology Institute, Eli and Edythe Broad Center of Regeneration Medicine and Stem Cell Research, and The Jonsson Comprehensive Cancer Center, David Geffen School of Medicine, University of California, Los Angeles, Los, Angeles, CA 90095

## Abstract

Neural stem cells (NSCs) can self-renew and give rise to the major cell types of the CNS. Studies of NSCs include the investigation of primary, CNS-derived cells as well as animal and human embryonic stem cell (ESC)-derived and induced pluripotent stem cell (iPSC)-derived sources. NSCs provide a means with which to study normal neural development, neurodegeneration, and neurological disease and are clinically relevant sources for cellular repair to the damaged and diseased CNS. Proteomics studies of NSCs have the potential to delineate molecules and pathways critical for NSC biology and the means by which NSCs can participate in neural repair. In this review, we provide a background to NSC biology, including the means to obtain them and the caveats to these processes. We then focus on advances in the proteomic interrogation of NSCs. This includes the analysis of posttranslational modifications (PTMs); approaches to analyzing different proteomic compartments, such the secretome; as well as approaches to analyzing temporal differences in the proteome to elucidate mechanisms of differentiation. We also discuss some of the methods that will undoubtedly be useful in the investigation of NSCs but which have not yet been applied to the field. While many proteomics studies of NSCs have largely catalogued the proteome or posttranslational modifications of specific cellular states, without delving into specific functions, some have led to understandings of functional processes or identified markers that could not have been identified via other means. Many challenges remain in the field, including the precise identification and standardization of NSCs used for proteomic analyses, as well as how to translate fundamental proteomics studies to functional biology. The next level of investigation will require interdisciplinary approaches, combining the skills of those interested in the biochemistry of proteomics with those interested in modulating NSC function.

Neural stem cells, which are present both during development and in the adult, are most commonly defined by the ability to self-renew and the capacity to generate the major cell types in the central nervous system (CNS)^1^, including oligodendrocytes, astrocytes, and neurons. Within this seemingly simple definition, however, the diversity of what is termed “neural stem cells” is quite large. There is a broad spectrum of NSCs with varying degrees of potency from multi- to more limited progenitors, each with unique lineages, fates, and spatial and temporal molecular signatures, that ultimately give rise to the vast numbers of mature CNS cell types (1–5). In this review, the term “NSC” will be used to generally describe this heterogeneous family of neural stem and progenitor cells. The study of NSCs has led to major advances in neural development and also to the vision of therapeutic uses in neurodegeneration, disease, and aging.

While the first evidence of proliferating cells within the human brain was found in the 1800s, the age of NSC studies began in earnest in the 1990s with the development of advanced techniques, including approaches to isolation and purification, *in vitro* models, lineage tracing, and molecular profiling (historical review (6)). As illustrated in Fig. 1, there are currently three primary means of obtaining NSCs: (1) direct isolation from the developing or adult CNS using a variety of markers; (2) amplification of isolated cells *in vitro*; and (3) directed differentiation from pluripotent cells consisting of either embryonic stem cells (ESCs) or induced pluripotent stem cells (iPSCs). Each of these approaches to isolation or enrichment comes with its own drawbacks. Ideally, all studies would use *bona fide* NSCs purified from *in vivo* sources. However, despite a great deal of effort, there are no protein markers that absolutely purify even one type of NSCs, perhaps a reasonable finding given NSC diversity. On the other hand, any tissue culture method being used will introduce both heterogeneity as well as tissue culture artifacts.

**Fig. 1.**
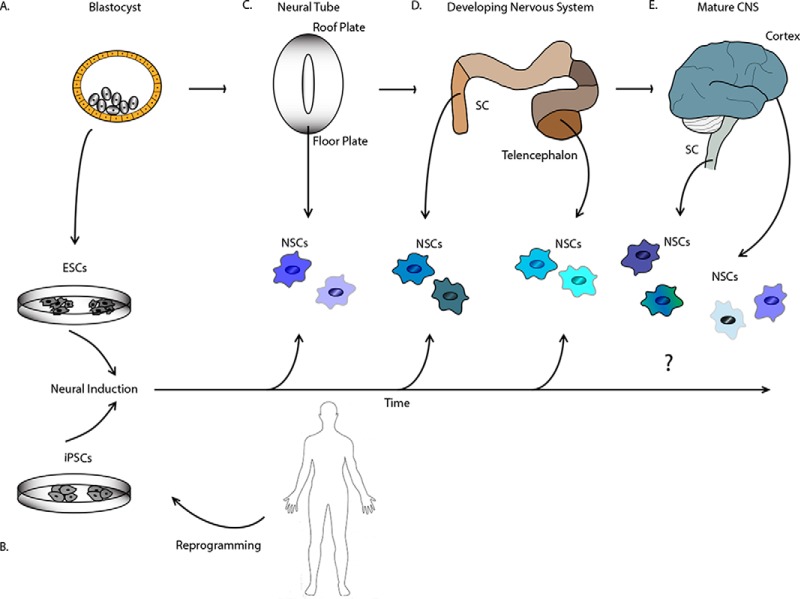
**Illustration of the primary means of obtaining neural stem cells (NSCs).** Pluripotent stem cells such as (*A*) embryonic stem cells (ESCs) obtained from the blastocyst or (*B*) induced pluripotent stem cells (iPSCs) derived from mature somatic cells can be directed to a neural stem cell fate in culture to generate NSCs. NSCs can also be isolated directly from (*C*) the developing neural tube, (*D*) the developing nervous system, or (*E*) from the adult CNS. The type of NSCs obtained is a function of both *in vivo* regional and temporal aspects as well as the *in vitro* environment the cells experience, as represented by the varying color and shape of the NSCs. CNS: central nervous system; SC: spinal cord. Not drawn to scale.

The first neural stem cells identified were *in vitro* models isolated from embryonic rat forebrain (7) and adult mouse brain (8). However, CNS-derived NSCs have now been obtained from a multitude of developmental stages and brain regions, including spinal cord, grown *in vitro* as attached or floating cultures, and exist as both primary and established cell lines (Figs. 1*C*, 1*D*, and 1*E*) (8–11). The isolation of pluripotent embryonic stem cells (ESCs) from mice (12, 13) and from humans (14) ushered in a new era for the field and has led to novel approaches to drive ESCs to a NSC fate (Fig. 1*A*) (15–17). Advances in understanding the maintenance of ESC pluripotency led directly to the manipulation of key molecules associated with ESC pluripotency, in particular octamer-binding transcription factor 4 (OCT4), (sex determining region Y)-box 2 (SOX2), Kruppel-like factor 4 (KFL4), and the transcription factor, c-MYC, to generate pluripotent iPSCs from fully differentiated somatic cells (18, 19). Approaches to generating iPSCs have evolved since the initial description and now include many innovations and much diversity in methodologies, including various cocktails of transcription factors, transcription factor delivery options, xeno-free cultures, and nonintegrating approaches (20) (reviewed in (21)). The generation of human iPSCs has led to technological advances in the study of NSCs (Fig. 1*B*) (22–24) and has had important implications for the realization of effective therapies, as significant differences exist between human and animal NSCs and disease.

## 

### 

#### Utility of NSCs

With the lofty goal of neural repair, there is clear potential for clinical uses of NSCs, either through transplantation of NSCs, of more committed cell types, of autologous iPSC-derived cells, or of manipulation of endogenous stem cells *in vivo*, for diseases ranging from ALS, Parkinson's disease, spinal cord injury, and stroke (reviewed in (25)). In the initial approaches of NSCs for use in neural repair, investigators were primarily focused on discovering the means to direct cell fate in a general fashion: that is, to guide cells to become either neurons, astrocytes or oligodendrocytes, or, at best, to guide cells to synthesize a specific neurotransmitter, such as dopamine for Parkinson's disease. Despite decades of successful research in the field (26, 27), many questions remain, including what defines NSCs molecularly, how this signature may be manipulated to produce certain cell types, how similar NSCs are when derived from different sources, what are the primary signaling pathways, master transcriptional controls, key protein posttranslational modifications and epigenetic changes, and what drives cell specification and differentiation *in vitro* and *in vivo*? However, as the knowledge of developmental biology and the technology of reprogramming have grown, simplistic approaches have given way to attempts to direct general cell fate in a more specific fashion. For instance, although clinical studies of differentiated iPSCs are in their infancy, a recent report detailed the transplantation of cortically fated neuronal progenitors derived from human iPSCs, resulting in mature, functional cortical neurons and functional recovery in a rodent stroke model (28). By being more sophisticated in (re)programming specific progenitors or cell types and by providing a finer molecular definition of those cells, we may be in a better position to affect and *direct* clinical outcomes, to improve efficacy and safety for future transplantation.

A major challenge lies in the understanding of the mechanisms of action of NSC transplants. It is unclear how NSC treatments might exert their benefit—will they be capable of generating complex circuitry, or will their effects be to provide general support for endogenous repair mechanisms? The existing theories for potential benefit of NSCs include the delivery of trophic support to the injured tissue, increased host cell survival, provision of immunomodulation, contributions to angiogenesis, and integration into the host tissue to provide cellular scaffolding and re-establishing synapses and neural circuits. To understand the *in vivo* consequences of NSC transplants, technological advances are necessary to address for instance the difficult task of distinguishing host *versus* donor tissue. In recent elegant work, Kumamaru *et al.* describe the *in vivo* isolation and RNA-seq profile of mouse spinal cord derived neural stem/progenitor cells (NSPCs) from host tissue following transplantation into a spinal cord injury model (29). This type of study has yet to be accomplished at the level of the proteome with current proteomics approaches, and remains a technological challenge.

Importantly, NSC research was previously dominated primarily by rodent-derived stem cells, but human ESC and in particular human iPSC technology has led to an increase in human-derived stem cell studies. iPSC technologies are increasingly being utilized to develop “disease-in-dish” approaches using patient-derived cells, offering a significantly more “human,” albeit *in vitro*, environment and have been used to study diseases as diverse as epilepsy, Huntington's disease, schizophrenia, and Parkinson's disease (reviewed in (30) and covered in greater detail in this special issue of *MCP*).

#### Neural Stem Cell Biology

Although a detailed review of NSC biology is beyond the scope of this review, we will briefly cover some basics to illustrate the diversity of NSCs present throughout development. The purpose here is to familiarize the nonexpert reader, who may be interested in interrogating NSC with proteomics, with the basics of the field. During embryonic and fetal development, NSCs are found throughout the neuraxis in specialized pseudostratified columnar neuroepthelium lining the surfaces of the developing ventricular system and central canal. During these stages, NSCs express the intermediate filament, nestin (31), and the extracellular protein, Lex/SSEA1/CD15 (32) and have a high proliferative and neuronogenic capacity. These cells are, in fact, radial glia (33), long thought to be mere scaffolds for neuronal migration, and have attachments extending from the ventricular to the pial (or outer) surfaces. As development proceeds, in most CNS regions, the ventricular zone thins. However, in the forebrain, some NSCs detach from the ventricular surface to form the subventricular zone, which then gives rise to an additional, even more superficial zone termed the outer radial glia zone (34), which also contains NSCs. Over the course of development within most brain regions, NSCs are first predominantly neuronogenic, generating primarily neurons, and later become largely gliogenic, a pattern that holds to some extent *in vitro*, with earlier derived NSCs giving rise mainly to neurons and later ones primarily to glia (35). During this time, NSCs generally switch their intermediate filament expression from nestin to glial fibrillary acidic protein (36–38) and are occasionally termed astrocytes, although astrocytes located in the parenchyma, away from these germinal zones do not have the characteristics of NSCs. In the adult mammalian brain, glial fibrillary acidic protein-positive NSCs are located in a thin subventricular zone as well as in the hippocampus, where they give rise to new granule cells that participate in the formation of certain memories (37). In the forebrain subventricular zone lining the lateral ventricle, NSCs give rise to olfactory bulb neurons that may participate in certain forms of olfactory learning and memory (39), although it is not clear whether this is the case in humans, as at least some subventricular zone NSCs participate in the genesis of neurons in the adjacent caudate nucleus (40).

In addition to these well-known NSC locations and sources, there are other, more poorly understood or less well-known regions harboring neural stem cells. Though controversial, there is a growing body of evidence suggesting that in some brain regions, ependymal cells (specialized ciliated cells lining the ventricular surfaces) also function as NSCs (41, 42). Furthermore, prior to the appearance of definitive, nestin-expressing neural stem cells, more primitive neural stem cells exist that have the capacity to give rise to neural stem cells but also may produce nonneural lineages when injected into blastocysts (43). Additionally, there are multipotent neural progenitors located at the surface of the cortex, in the marginal zone, far removed from the traditional germinal zones during development (44).

NSCs from different brain regions and developmental stages possess distinct characteristics, as illustrated in Figs 1*C*-1*E*. This includes intrinsic neuronogenic and gliogenic capacity but also dorsal-ventral patterning signatures and many other qualities. Furthermore, within a distinct brain region, there may be multiple types of cells with the characteristics of NSC self-renewal and multipotency. Indeed, the act of placing cells in culture may obscure some of these differences. For example, oligodendrocytes are normally specified from select brain regions during development, but *in vitro*, all NSCs are capable of giving rise to oligodendrocytes. To further complicate the situation, cells that behave only as short-term progenitors *in vivo* may behave as long-, self-renewing NSCs *in vitro*(45).

#### NSC Methodologies

There has been a large effort to develop reliable systems to purify neural stem cells from living tissue for biochemical analysis. Early research focused on standard FACS methodologies using combinations of cell size and protein expression (46) as well as dye exclusion based on expression of ABC transporters (47). Other strategies have included the use of promoter-specific expression (48, 49) or extracellular markers such as 3-fucosyl-N-acetyl-lactosamine (or SSEA1, CD15, or LeX) (50) in mice and prominin1 (or CD133) in humans (51). These methods, while producing some degree of enrichment, have not necessarily resulted in the isolation of a pure stem cell fraction. More recently, investigators have used a combination of methods, including transgenic mice and extracellular promoters to provide a greater level of enrichment (52, 53). Even given these improvements, obtaining sufficient quantities of a pure population of NSCs for a comprehensive proteomic study can present significant challenges.

NSCs can be cultured in a variety of ways. They can be easily propagated from the developing and adult CNS using a simple serum-free medium supplemented with basic fibroblast growth factor (FGF) and/or epidermal growth factor (EGF). In early rodent embryonic development, isolated NSCs respond only to basic fibroblast growth factor and not to EGF, although postnatally derived NSCs respond to both (54). NSCs can be cultured as nonattached neurospheres (NS) or as adherent monolayers and can now be derived from both the developing and adult human CNS, although the survival and growth of adult NSCs are improved with the addition of leukemia inhibitory factor (55). The culturing of NSCs can greatly expand the numbers of cells for subsequent studies, but under no circumstances are these cultures homogeneous. To partially mitigate this, comparative studies involving, for instance, proliferating cultures with those induced to differentiate are a valuable approach. We have taken advantage of this contrast to examine both mRNA (56) and protein expression (57) in NSCs, identifying genes and proteins with roles in NSC biology such as maternal embryonic leucine zipper kinase (MELK), t-lak cell-originated protein kinase (TOPK). and the netrin/repulsive guidance molecule (RGM) receptor, neogenin.

The strength of using *in vitro* systems may lie in the ability to model human development and disease. Therefore, a great deal of effort has been placed in the development of regional and cell-type specific production of neural progenitors from human pluripotent stem cells. Human pluripotent stem cells (ESC and iPSC) can serve as a reservoir to generate large numbers of NSCs. Recent years have seen an explosion in the methodologies to accomplish this. ESCs can be manipulated via a myriad of morphogens to produce a variety of types of NSCs, including spinal motor progenitors, cerebral cortical excitatory and inhibitory neuronal progenitors, midbrain dopamine progenitors, and glial progenitors. Obtaining large numbers of progenitors from mouse ESCs is relatively rapid and easy. Most protocols now involve generation of floating embryoid bodies followed by replating until they form neural “rosettes,” structures that bear some resemblance to the neural tube (58, 59). The rosettes, which contain the primitive NSC-like cell described above, are then manually picked, replated, and ultimately propagated as NSCs. Using these fundamental methods, the production of various types of neurons can be manipulated using specific factors to direct this differentiation. For example, there are factors that caudalize and ventralize the NSCs to produce those with the characteristics of spinal cord motor neurons (60). Interestingly, apparent cerebral cortical stem cells and then cortical excitatory neurons can be generated simply by omission of these caudalizing or ventralizing signals (61). The drawbacks to using any of these *in vitro* methods, again includes the heterogeneity of the cultures and, in the case of human cells, the considerable time and expense required to produce sufficient numbers of cells for subsequent analyses. Another drawback to the process lies in the lack of complete knowledge as to how the developmental stage of pluripotent stem cell-generated NSCs and their progeny correspond to those of the true CNS *in vivo*. A great deal of effort is now being placed on developing tools to ascertain this relationship (62, 63); however, the continuing development of methodologies to derive region-specific NSCs will allow greater accuracy and relevance of all “omics” studies, including those of proteomics. In addition, as limits of detection for proteomics and other “omics” studies continue to improve, the amount of starting material required for various analyses continues to decrease, enabling the interrogation of smaller populations of cells.

#### Interrogating NSCs—Why Proteomics?

Numerous studies have investigated the transcriptomics of neural stem cells and the process of neural stem cell differentiation and cell fate, an example of which is described above, in which we used differential mRNA expression to identify key regulators of neural stem cell proliferation (56). These studies have contributed to the understanding of many facets of neural stem cell biology and have identified key regulators of neural stem cell function. While RNA-based approaches have a large part to play in delineating the roles of specific proteins in neural stem cell function, there is an important role for proteomics to play as well. Not only do proteomics studies help to provide a “fuller” picture of the actual state of cells than does simple mRNA profiling, because gene expression does not always correlate with protein expression (64), but there are also important facets of neural stem cell biology that can really only be fully ascertained by proteomic analyses. Proteomics studies are particularly well-suited to identify markers of NSC in all their varying forms, as well as to identify secreted proteins that endogenous and transplanted neural stem cells may produce. Also, since many of the pathways involved in cell fate determination are activated or inhibited by posttranslational modifications such as phosphorylation or ubiquitination, it is only through proteomics studies that we will gain an understanding of these processes. Furthermore, proteomics analyses can be used to determine subcellular localizations of proteins, information that can only be partially inferred from RNA-based methods. Excellent reviews on various aspects of stem cell proteomics have been recently published (65–67).

In the following sections, we have highlighted a number of studies to illustrate the potential of proteomics to address the main questions of characterization of the proteins of NSCs, the location of those proteins in NSCs, protein expression changes over time and with differentiation, and finally, the molecular differences between NSCs and their parents and progeny. While the studies presented demonstrate a start, the field is still in its infancy and few studies have made the jump from cataloguing proteins to analyzing function. A great deal more research is needed to bring proteomics to the forefront of NSC research.

#### Characterization of NSC Protein Expression

Some of the first studies to establish protein signatures of various NSCs addressed the very basic but essential question of the composition of these cells and often used the most available platforms—2 dimensional gel electrophoresis (DGE) and MALDI-TOF MS—to analyze, for instance, rat hippocampal NSCs (68) or a human NSC line (69). At the time, MS approaches were an emerging technology with 500–1,200 proteins detected and anywhere from 200–400 actually identified. Seemingly continual advances in separation technology, label-based or label-free quantitation, single or multiple reaction monitoring, detection, instrumentation, and software have led to a massive leap in the number of proteins observed and confidently identified—routinely surpassing thousands in one experiment—and it is estimated that greater than 90% of the human proteome has now been defined according to ProteomicsDB (70). The result is a vast improvement on the “resolution” of the proteome profile (for an overview see (71, 72)).

Posttranslational modifications (PTMs) are an important component of NSC characterization and provide insight into cellular signaling, differentiation, and maintenance. MS is particularly amenable to the large-scale analysis of PTMs, modifications that are not easily predicted by other techniques, particularly as advanced PTM enrichment strategies are developed, as proteome coverage is increased, and as MS techniques advance (reviewed in (73)). Phosphorylation, glycosylation, acetylation, and ubiquitination are some of the more common PTMs, but there are well over 300 different types of PTMs, including those affecting epigenetic changes such as acetylation and methylation modifications of histones (reviewed in (67, 74)). Despite the technological challenges of large-scale PTM studies, often including the reliance on PTM enrichment, there has been a number of key studies. Differences in the phosphoproteomes of human undifferentiated and retinoic acid (RA)-differentiated (neuralized) ESCs were identified and resulted in the identification of hundreds of proteins with unique phosphorylation (75). There were multiple altered signaling pathways as ESCs acquired a neural fate following treatment with RA, including a previously undescribed role for the JNK pathway. To gain insight into global ubiquitination—termed the “ubiquitome”—Buckley *et al.* used stable isotope labeling by amino acids in cell culture (SILAC) and ubiquitinated peptide enrichment to identify modified proteins from RA-mediated differentiated and pluripotent mouse ESCs and iPSCs (76). The authors identified 17 genes that regulated ESC differentiation toward a neural fate, three of which they validated (*Fbxw7*, *Socs3*, and *RNF31*). In a major effort to understand the quantitative changes in the membrane proteome during differentiation of human ESCs to NSCs, Nunes Melo-Braga and colleagues isolated membrane-bound proteins from both cell populations, further identified specific multi- and mono-phosphorylation and sialic acid *N*-linked glycosylation PTMs, and validated several proteins with modified sites by selected reaction monitoring (77). In addition to providing a wealth of data on membrane-bound proteins, including the identification of novel potential surface markers of NSCs such as Crumbs 2 (which was up-regulated at the both the proteome and sialiome levels), this group identified unique glycosylation patterns in both cell populations and highlighted the potential importance of sialylated glycoproteins in the definition of the stem cell state.

In an extension of this approach to examine multiple PTMs, PTM “crosstalk” is a growing field that focuses not on global PTMs but rather where the PTMs are occurring on the protein and how these PTMs relate to each other to alter downstream events such as signaling or transcription (reviewed in (78)). While this has yet to be applied to NCSs on a large scale, an example of how such a method might be used is provided by Jung *et al.* who used weak cation exchange and hydrophilic interaction liquid chromatography to separate 5–6 kDa N-terminal tail H3 histone peptides from mouse ESCs followed by high mass accuracy electron transfer dissociation MS/MS to identify 114 peptides with unique H3 methylation, acetylation, and phosphorylation patterns (79). They went on to produce a combinatorial map of the histone PTM landscape that may potentially be ESC-specific, thereby gaining insight into the pluripotent state.

#### Comparative Expression

To develop a greater understanding of the mechanisms underlying NSC biology and to address heterogeneity in NSC populations, many studies have focused on identifying differentially expressed proteins or PTMs. Earlier studies relied on 2DGE methodology as a means to compare samples, as was the case to identify membrane-associated proteins that may play a role in maintaining a more potent state in the mouse embryonic NSPCs as compared with those isolated from the postnatal brain (57). The development of protein labeling techniques, in particular SILAC and isobaric tags such as iTRAQ, has allowed mass spectrometry to enter the realm of relative and absolute quantification. This was demonstrated in the use of SILAC to compare the proteome and phosphoproteome of two human ESC lines, as well as the complement of membrane proteins specific to undifferentiated and to spontaneously differentiated ESCs upon removal of FGF (80). The group identified six membrane proteins specifically up-regulated in hESCs, including Prominin-1(a known ESC marker) and Neuroligin-4, and 17 membrane proteins whose expression was increased during differentiation that included matrix metallopeptidase (MMP)-14 and Semaphorin 4A. While the ESCs were nonspecifically differentiated to NSCs in this study, using this approach in a more detailed differentiation of ESCs along the neural lineage could provide additional membrane protein candidates that could be used for subsequent purification of subsets of neural-fated progenitors. To address a clinically relevant question regarding the differences between motoneurons (MN) derived from iPSCs and from ESCs, Toma *et al.* used FACS to purify MN generated from both iPSCs and ESCs that expressed green fluorescent protein under a motoneuron promoter, Hb9 (81). Using a label-free LC-MS/MS analysis, they identified 3,025 proteins, 50 of which were unique to iPSCMNs and 93 to ESCMNs, and 28 proteins up-regulated and 87 downregulated in iPSCMNs compared with ESCMNs. The relatively small number of differentially expressed proteins did not result in any significant differences in experimental outcome between these two sources of MN *in vitro* or *in vivo*, suggesting that iPSC-derived MN may indeed be a suitable alternative to ESCMNs in therapeutic and pharmaceutical testing applications.

#### Spatial Aspects of Expression

Subcellular fractionation followed by MS-based identification allows the detailed study of cellular compartments. In the context of NSCs, identification of secreted proteins may lead to development of better hypotheses on mechanisms of action whereas identification of membrane proteins may shed light on potential signaling cascades and also inform novel means to further purify subpopulations. Not to ignore the intracellular compartments, understanding where proteins reside at a certain time also reveals information on cell states, for instance the sequestration of transcription factors in the cytoplasm limits physical access of the transcription factors to regulate transcription in the nucleus.

A highly supported hypothesis regarding mechanism of action of stem cells in transplantation is the provision of trophic factors to the injured environment. Obtaining those species secreted by NSCs *in vitro* into the culture medium is relatively easy but is a challenging proteome to analyze, given the dynamic range of protein expression and the often necessary addition of serum or supplements to the medium for normal cell growth, which can further complicate the identification of low abundance proteins. One group used an immortalized mouse NSC line derived from embryonic mesencephalon in an effort to identify secreted proteins as NSCs differentiate, first using LC-MS/MS (82) and then with a targeted array of proteins using a multiplexed immunoassay (83). Using MS, 104 nonredundant proteins were detected such as secreted protein, acidic, cysteine-rich (SPARC), neural cell adhesion molecule (NCAM), and pigment epithelium-derived factor (PEDF), but the list was likely heavily biased toward the most abundant members of the secretome, which included fibronectin, vimentin, alpha-actinin, and heat shock protein 90 (HSP90). To address the issues of protein dynamic range, they then turned to a commercially available targeted multiplexed immunoassay to detect expression of 23 cytokines and chemokines that were predicted to be present at much lower levels and identified a potential role for the chemokine (C-C motif) ligand 2 (CCL2 or MCP) in neural differentiation. Similarly, a commercially available array targeting 507 proteins was used to screen the secretome of a human NSC line compared with the same line that had been differentiated into oligodendrocytes by overexpression of the transcription factor, *Olig2* (84). The authors then combined these data with RNA-seq data from the same populations to discover many novel ligand-receptor pairs coexpressed by oligodendrocytes, such as chemokine (C-X-C motif) receptor 2 (CXCR2) and its ligands, chemokine (C-X-C motif) ligand (CXCL) 1–3, CXCL5, CXCL6, and CXCL8, and the interleukin-6 (IL-6) ligand and its receptors interleukin-6 receptor (IL6R) and interleukin-6 signal transducer (IL6ST), suggesting autocrine signaling may play a more important role in the differentiation and maintenance than previously thought. To gain insight into the temporal aspects of the differentiating secretome, Farina *et al.* examined mouse ECSs to those acquiring a cardiac and a neural fate during early and late commitment and identified a number of secreted proteins unique to the neurogenic group such as thrombospodin-1, ezrin, and biglycan (85). A review of additional secretome studies can be found in (86).

A less well-studied “omics,” lipidomics, is defined as the large-scale analysis of membrane lipids. Lipids are able to both directly and indirectly modulate protein function and consist of several categories, including sphingolipids, glycerolipids, glycerophospholipids, fatty acids, and sterols. Lipids are known to have key roles in NSC biology. For instance, lysophosphatidic acid is capable of regulating proliferation of mouse NSCs in a neurosphere model (87) and of the differentiation to neural lineages of human NSCs derived from ESCs (88). One of the first studies to use advanced, large-scale MS techniques involved the profiling of retinal stem cells and demonstrated a retinal stem cell-unique signature compared with non-retinal stem cells (89). This category of molecules clearly has an important role in stem cell biology, yet there have been very few studies directed at understanding the landscape of lipids within NSCs. Improvements in lipidomics platforms, such as that recently reported by Almeida *et al.* to quantitatively identify over 300 lipid species in mouse brain tissue, may change that (90).

#### Temporal Aspects of Expression

One of the pressing issues in the field is the elucidation of molecular routes to differentiation to better understand the underlying mechanisms of neural development and to better direct cellular outcomes (reviewed in (67, 91)). In two separate efforts, Chaerkady and colleagues studied temporal quantitative changes in protein expression at eight different time points as human ESCs were differentiated to neural progenitor cells and into motor neurons and astrocytes using 8-plex iTRAQ and quadrupole time-of-flight (92) and into glial and oligodendrocyte progenitor lineages using 4-plex iTRAQ and LTQ-Orbitrap (93). The subsequent identification of over 4,000 proteins represents a rich source of temporally and lineage-specific proteins for further study. Focusing on the membrane proteome changes during glial differentiation, Cao *et al.* used an immortalized mouse neural stem cell line to identify membrane proteins expressed by the undifferentiated neural stem cell and following astrocyte differentiation using SILAC labeling and LTQ-Orbitrap (94). Out of the more than 700 proteins identified in this study, 205 were differentially expressed by 1.5-fold or more, with the additional finding that changes in expression of mitochondrial membrane proteins was associated with glial differentiation. To demonstrate the power of combining discovery-based proteomics with multiple reaction monitoring, Yocum and colleagues studied feeder cell- and conditioned medium-free human ESCs following the acquisition of a neural fate via modulation of bone morphogenic protein signaling (95). Using the iTRAQ platform, quantitative global protein expression was accomplished using the iTRAQ platform, followed by multiple reaction monitoring of a selection of proteins to both validate the global proteomics results and to quantitate protein isoforms by utilizing isoform-unique peptides.

With so many groups studying various aspects of stem cells in general, efforts are underway to facilitate collaboration, and the sharing of data and methodology. The Human Proteome Organization (HUPO) and the International Society for Stem Cell Research (ISSCR) jointly established the Proteome Biology of Stem Cells Initiative, which serves at an international level to move the field of stem cell proteomics forward and to promote collaboration among international research groups. Online repositories and resources also exist, each with various strengths and data foci, a few of which include scor.chem.wisc.edu, proteomicsdb.org, proteomexchange.org, stemformatics.org, ebi.ac.uk/pride, and stemcelldb.nih.gov (96). A useful review of major proteomics repositories and databases can be found in (97).

#### Emerging Technologies

#### Software and Bioinformatics

NSCs are increasingly being defined by a multitude of “omics” approaches—genomic, transcriptomic, translatomic, and epigenomic as well as proteomic—each with varying degrees of technological maturity. Proteomics data are often going hand-in-hand with other larger datasets such as chromatin remodeling, RNA-seq, and immune profiling to name a few. These large datasets present both tremendous promise and challenge. Data *integration*, as opposed to data addition, becomes an issue for a number of reasons—not only is the information from each platform unique, but it also represents distinctive datasets and organization, containing fundamentally disparate data with differing limitations, and false discovery rates. Getting these datasets data to “talk” requires some level of programming bridging and is becoming a much more crucial component of analyses as researchers go beyond the classic single methodology approach. A major tour-de-force effort recently examined the genome-wide CpG (-C-phosphate-G- regions of the DNA) methylation and chromatin marks, the transcriptome (miRNA, lncRNA, mRNA), and the global and cell-surface quantitative proteome of iPSC reprogramming, the data resulting from which can be found at www.stemformatics.org through the appropriately named Project Grandiose portal (98). The means with which to accumulate these large datasets for easy public access, along with the tools with which to query it, continue to be developed. Groups, such as those behind Stemformatics, are further establishing repositories of data and tools to facilitate the analysis, visualization, sharing, and access of stem cell data (99).

#### Novel Platforms for NSC Research

In addition to the commonly used MS-based platforms, there are some novel techniques being developed for targeted proteomic studies that have yet to be applied to NSCs and present opportunities for future research. Single-cell mass cytometry makes use of the combination of stable nonbiological isotope tagged antibodies with inductively coupled plasma mass spectrometry detection, currently allowing over 40 different parameters to be analyzed simultaneously per cell at a rate of about 500 cells per second (100). Its potential was recently demonstrated in the profiling over time of the generation of iPSCs from fibroblasts via three separate reprogramming protocols, generating a single cell comprehensive map of the conversion somatic cells to a pluripotent state (101). One of the advantages to this technique is the single cell resolution, currently challenging using other mass-spectrometry-based systems, which is particularly important when mapping out differentiation programs or understanding the heterogeneity in a cell population.

The generation of high-quality human antibodies through the efforts of the Human Protein Atlas has moved affinity proteomics to another level (102, 103). Using highly multiplexed antibody bead arrays as means to identify neurological disease, Byström *et al.* systematically screened plasma samples of patients with multiple sclerosis with over 4,595 antibodies to ultimately focus the effort to the expression of 43 proteins using 101 antibodies on larger numbers of samples of both plasma and CSF (104). Confirmation of multiple sclerosis specific protein expression was made in human post-mortem tissue and plasma and has provided a number of potential circulating protein targets and their respective neural cell type source. This approach may prove to be powerful in elucidating mechanisms of action of NSC transplants, although the success of these studies clearly relies heavily on the quality of the available antibodies and the ability to confirm the results using other techniques.

A recently developed technique called comprehensive identification of RNA-binding proteins by mass spectrometry, or ChIRP-MS, allows the analysis of the less studied subproteome of RNA-binding proteins (105). Long noncoding RNAs (lncRNAs) have been shown to have key roles in neural development and NSC biology, including the identification of lncRNAs specific to NSCs in the adult mouse (106) and the expression of lncRNAs in the differentiation of embryonic NSCs into neuronal and glial lineages (107). lncRNAs exert control over transcription via RNA-binding proteins but the identification of these proteins with high specificity and comprehensive coverage has previously been technologically challenging. ChIRP-MS was used to identify 81 RNA-binding proteins specific to Xist, an lncRNA involved in X chromosome inactivation, via extensive crosslinking of the protein–RNA complex, followed by identification of those binding partners by LC-MS/MS (105). To identify proteins binding to key transcriptional enhancers, promoters, and chromatin in mouse ESCs, Engelen *et al.* used chromatin immunoprecipitation followed by mass spectrometry, a technique termed ChIP-MS (108). Using gel chromatography and an LQT-Orbitrap MS, 239 proteins were reported, comprising a rich set of candidates not only to understand the pluripotent state of ESCs but also to potentially identify new candidates in reprogramming of somatic cells to iPSCs. There is an opportunity to apply these approaches to NSCs and their progeny.

#### Challenges

Are there common NSC-specific themes or biological pathways that have emerged from the large body of existing research, including that provided through other technologies? In the RNA expression array field, this goal seems elusive (109) and is not likely realistic in the proteomics field, particularly given the increasingly diverse populations of NSCs. Indeed, a meta-analysis of published proteomics data from three unique human ESC lines identified only 32 intracellular and 16 membrane proteins in common (110). Cellular heterogeneity will continue to be an issue as there are a myriad of variables present up to the final dataset, including animal versus human cell sources, diversity of protocols for isolation, derivation and differentiation, introduction of artifacts through the use of feeder cells, or conditioned medium. Finally, there is a massive library of methodological and instrumental choices in the separation, enrichment, identification, and analysis steps. MS-based platforms continue to improve, and future experiments will benefit by further increasing the coverage of the proteome, from a cellular and subcellular point of view.

As outlined above, there are a variety of cells within the CNS that can be defined as NSCs. These are different according to CNS region and developmental timing and even within small brain regions; there may be multiple cell types that possess NSC characteristics. Although novel platforms are enabling enrichment of these NSC types, the yield of these sorting experiments may be extremely low and require a large amount of starting tissue to produce enough cellular material for proteomics experiments. Currently, the most common approach to obtain large quantities of NSCs is to rely on *in vitro* culturing. However, this has several drawbacks. In the case of derivation of NSCs from murine or human CNS, cultures undergo some degree of spontaneous differentiation, resulting in cellular heterogeneity. In fact, the simple act of culturing NSCs alters their qualities and characteristics by removing crucial *in vivo* spatial and temporal cues. As reviewed above, recent studies have greatly enhanced our ability to obtain region- and cell-type-specific NSCs from human and murine pluripotent stem cells. However, these NSCs may be different from those found *in vivo*. Furthermore, *in vitro* cultures require significant time and expense to reach the point of NSC generation for proteomic analyses, particularly in the case of human cells, and all cultures, including those pluripotent stem cell derived, will be somewhat heterogeneous. The caveats described above should not preclude proteomics studies of NSCs but rather suggest that all studies be done with caution and that all analyses be confirmed using anatomical and functional methods wherever possible. For example, if a proteomics experiment identifies a protein as being enriched in cultures of NSC derived from the cerebral cortex, the experimenters may then use immunohistochemistry to evaluate the localization of that protein *in vivo* and examine the function in more detail utilizing knockdown or other means, as we have reported (57).

The application of proteomics platforms has been crucial to our growing but incomplete understanding of the complex dynamics of how NSC fate is directed, how NSCs retain their potency and ability to self-renew, how NSCs populations differ, and finally how specific differentiation of NSCs can be accomplished. Proteomics approaches complement data obtained via other technologies, and it is the combination of these various datasets, obtained from well-defined populations of NCSs, that will continue to move the field ahead. The next stage of NSCs proteomics will require a multidisciplinary approach, combining the interests of biochemists, neuroscientists, and bioinformatics experts, and will allow proteomics studies to move from being technical achievements of interest to being a part of the routine armamentarium of those studying NSC cell biology, much as has occurred with studies of genomics and mRNA expression a few decades ago.

## Footnotes

^1^ The abbreviations used are:

NSCs: neural stem cells
CNS: central nervous system
ESCs: embryonic stem cells
iPSCs: induced pluripotent stem cells
NSPCs: neural stem/progenitor cells
MN: motoneurons
FACS: fluorescent activated cell sorting
RA: retinoic acid
FGF: fibroblast growth factor
EGF: epidermal growth factor
CpG: -C-phosphate-G-ChIRP-MS comprehensive identification of RNA-binding proteins by mass spectrometry
lncRNA: long noncoding RNA
ChIP-MS: chromatin precipitation mass spectrometry.

## References

[1] Molyneaux B. J., Arlotta P., Menezes J. R., and Macklis J. D. (2007) Neuronal subtype specification in the cerebral cortex. Nat. Rev. Neurosci. 8, 427–437 17514196 10.1038/nrn2151

[2] Zuchero J. B., and Barres B. A. (2013) Intrinsic and extrinsic control of oligodendrocyte development. Curr. Opin. Neurobiol. 23, 914–920 23831087 10.1016/j.conb.2013.06.005 PMC4431975

[3] Shoemaker L. D., and Arlotta P. (2010) Untangling the cortex: advances in understanding specification and differentiation of corticospinal motor neurons. BioEssays 32, 197–206 20108227 10.1002/bies.200900114 PMC4240621

[4] Guérout N., Li X., and Barnabé-Heider F. (2014) Cell fate control in the developing central nervous system. Exp. Cell Res. 321, 77–83 24140262 10.1016/j.yexcr.2013.10.003

[5] Sloan S. A., and Barres B. A. (2014) Mechanisms of astrocyte development and their contributions to neurodevelopmental disorders. Curr. Opin. Neurobiol. 27, 75–81 24694749 10.1016/j.conb.2014.03.005 PMC4433289

[6] Breunig J. J., Haydar T. F., and Rakic P. (2011) Neural stem cells: Historical perspective and future prospects. Neuron 70, 614–625 21609820 10.1016/j.neuron.2011.05.005 PMC3225274

[7] Temple S. (1989) Division and differentiation of isolated CNS blast cells in microculture. Nature 340, 471–473 2755510 10.1038/340471a0

[8] Reynolds B. A., and Weiss S. (1992) Generation of neurons and astrocytes from isolated cells of the adult mammalian central nervous system. Science 255, 1707–1710 1553558 10.1126/science.1553558

[9] Palmer T. D., Ray J., and Gage F. H. (1995) FGF-2-responsive neuronal progenitors reside in proliferative and quiescent regions of the adult rodent brain. Mol. Cell. Neurosci. 6, 474–486 8581317 10.1006/mcne.1995.1035

[10] Ma W., Fitzgerald W., Liu Q. Y., O'Shaughnessy T. J., Maric D., Lin H. J., Alkon D. L., and Barker J. L. (2004) CNS stem and progenitor cell differentiation into functional neuronal circuits in three-dimensional collagen gels. Experimental Neurology 190, 276–288 15530869 10.1016/j.expneurol.2003.10.016

[11] Kelly T. K., Karsten S. L., Geschwind D. H., and Kornblum H. I. (2009) Cell lineage and regional identity of cultured spinal cord neural stem cells and comparison to brain-derived neural stem cells. PLoS ONE 4, e4213 19148290 10.1371/journal.pone.0004213 PMC2615219

[12] Evans M. J., and Kaufman M. H. (1981) Establishment in culture of pluripotential cells from mouse embryos. Nature 292, 154–156 7242681 10.1038/292154a0

[13] Martin G. R. (1981) Isolation of a pluripotent cell line from early mouse embryos cultured in medium conditioned by teratocarcinoma stem cells. Proc. Natl. Acad. Sci. U.S.A. 78, 7634–7638 6950406 10.1073/pnas.78.12.7634 PMC349323

[14] Thomson J. A., Itskovitz-Eldor J., Shapiro S. S., Waknitz M. A., Swiergiel J. J., Marshall V. S., and Jones J. M. (1998) Embryonic stem cell lines derived from human blastocysts. Science 282, 1145–1147 9804556 10.1126/science.282.5391.1145

[15] Reubinoff B. E., Itsykson P., Turetsky T., Pera M. F., Reinhartz E., Itzik A., and Ben-Hur T. (2001) Neural progenitors from human embryonic stem cells. Nat. Biotechnol. 19, 1134–1140 11731782 10.1038/nbt1201-1134

[16] Zhang S.-C., Wernig M., Duncan I. D., Brustle O., and Thomson J. A. (2001) In vitro differentiation of transplantable neural precursors from human embryonic stem cells. Nat. Biotechnol. 19, 1129–1133 11731781 10.1038/nbt1201-1129

[17] Bain G., Kitchens D., Yao M., Huettner J. E., and Gottlieb D. I. (1995) Embryonic stem cells express neuronal properties in vitro. Dev. Biol. 168, 342–357 7729574 10.1006/dbio.1995.1085

[18] Gurdon J. B. (1962) The developmental capacity of nuclei taken from intestinal epithelium cells of feeding tadpoles. J. Embryol. Exp. Morphol. 10, 622–640 13951335

[19] Takahashi K., and Yamanaka S. (2006) Induction of pluripotent stem cells from mouse embryonic and adult fibroblast cultures by defined factors. Cell 126, 663–676 16904174 10.1016/j.cell.2006.07.024

[20] Yu J., Hu K., Smuga-Otto K., Tian S., Stewart R., Slukvin I. I., and Thomson J. A. (2009) Human induced pluripotent stem cells free of vector and transgene sequences. Science 324, 797–801 19325077 10.1126/science.1172482 PMC2758053

[21] Lowry W. E., and Plath K. (2008) The many ways to make an iPS cell. Nat. Biotechnol. 26, 1246–1248 18997764 10.1038/nbt1108-1246

[22] Park I.-H., Zhao R., West J. A., Yabuuchi A., Huo H., Ince T. A., Lerou P. H., Lensch M. W., and Daley G. Q. (2008) Reprogramming of human somatic cells to pluripotency with defined factors. Nature 451, 141–146 18157115 10.1038/nature06534

[23] Takahashi K., Tanabe K., Ohnuki M., Narita M., Ichisaka T., Tomoda K., and Yamanaka S. (2007) Induction of pluripotent stem cells from adult human fibroblasts by defined factors. Cell 131, 861–872 18035408 10.1016/j.cell.2007.11.019

[24] Yu J., Vodyanik M. A., Smuga-Otto K., Antosiewicz-Bourget J., Frane J. L., Tian S., Nie J., Jonsdottir G. A., Ruotti V., Stewart R., Slukvin I. I., and Thomson J. A. (2007) Induced pluripotent stem cell lines derived from human somatic cells. Science 318, 1917–1920 18029452 10.1126/science.1151526

[25] Aboody K., Capela A., Niazi N., Stern J. H., and Temple S. (2011) Translating stem cell studies to the clinic for CNS repair: Current state of the art and the need for a Rosetta stone. Neuron 70, 597–613 21609819 10.1016/j.neuron.2011.05.007

[26] Greig L. C., Woodworth M. B., Galazo M. J., Padmanabhan H., and Macklis J. D. (2013) Molecular logic of neocortical projection neuron specification, development and diversity. Nat. Rev. Neurosci. 14, 755–769 24105342 10.1038/nrn3586 PMC3876965

[27] Franco S. J., and Müller U. (2013) Shaping our minds: stem and progenitor cell diversity in the mammalian neocortex. Neuron 77, 19–34 23312513 10.1016/j.neuron.2012.12.022 PMC3557841

[28] Tornero D., Wattananit S., Grønning Madsen M., Koch P., Wood J., Tatarishvili J., Mine Y., Ge R., Monni E., Devaraju K., Heverner R. F., Brüstle O., Lindvall O., and Kokaia Z. (2013) Human induced pluripotent stem cell-derived cortical neurons integrate in stroke-injured cortex and improve functional recovery. Brain 136, 3561–3577 24148272 10.1093/brain/awt278

[29] Kumamaru H., Ohkawa Y., Saiwai H., Yamada H., Kubota K., Kobayakawa K., Akashi K., Okano H., Iwamoto Y., and Okada S. (2012) Direct isolation and RNA-seq reveal environment-dependent properties of engrafted neural stem/progenitor cells. Nat. Commun. 3, 1140 23072808 10.1038/ncomms2132

[30] Han S. S., Williams L. A., and Eggan K. C. (2011) Constructing and deconstructing stem cell models of neurological disease. Neuron 70, 626–644 21609821 10.1016/j.neuron.2011.05.003

[31] Lendahl U., Zimmerman L. B., and McKary R. D. (1990) CNS stem cells express a new class of intermediate filament protein. Cell 60, 585–595 1689217 10.1016/0092-8674(90)90662-x

[32] Capela A., and Temple S. (2006) LeX is expressed by principle progenitor cells in the embryonic nervous system, is secreted into their environment and binds Wnt-1. Dev. Biol. 291, 300–313 16458284 10.1016/j.ydbio.2005.12.030

[33] Noctor S. C., Flint A. C., Weissman T. A., Dammerman R. S., and Kriegstein A. R. (2001) Neurons derived from radial glial cells establish radial units in neocortex. Nature 409, 714–720 11217860 10.1038/35055553

[34] Hansen D. V., Lui J. H., Parker P. R., and Kriegstein A. R. (2010) Neurogenic radial glia in the outer subventricular zone of human neocortex. Nature 464, 554–561 20154730 10.1038/nature08845

[35] Qian X., Shen Q., Goderie S. K., He W., Capela A., Davis A. A., and Temple S.(2000) Timing of CNS cell generation: A programmed sequence of neuron and glial cell production from isolated murine cortical stem cells. Neuron 28, 69–80 11086984 10.1016/s0896-6273(00)00086-6

[36] Doetsch F., Caillé I., Lim D. A., García-Verdugo J. M., and Alvarez-Buylla A. (1999) Subventricular zone astrocytes are neural stem cells in the adult mammalian brain. Cell 97, 703–716 10380923 10.1016/s0092-8674(00)80783-7

[37] Garcia A. D., Doan N. B., Imura T., Bush T. G., and Sofroniew M. V. (2004) GFAP-expressing progenitors are the principal source of constitutive neurogenesis in adult mouse forebrain. Nat. Neurosci. 7, 1233–1241 15494728 10.1038/nn1340

[38] Imura T., Kornblum H. I., and Sofroniew M. V. (2003) The predominant neural stem cell isolated from postnatal and adult forebrain but not early embryonic forebrain expresses GFAP. J. Neurosci. 23, 2824–2832 12684469 10.1523/JNEUROSCI.23-07-02824.2003 PMC6742109

[39] Gregorian C., , Nakashima J., Le, Belle J., Ohab J., Kim R., Liu A., Smith K. B., Groszer M., Garcia A. D., Sofroniew M. V., Carmichael S. T., Kornblum H. I., Liu X., and Wu H. (2009) Pten deletion in adult neural stem/progenitor cells enhances constitutive neurogenesis. J. Neurosci. 29, 1874–1886 19211894 10.1523/JNEUROSCI.3095-08.2009 PMC2754186

[40] Ernst A., Alkass K., Bernard S., Salehpour M., Perl S., Tisdale J., Possnert G., Druid H., and Frisén J. (2014) Neurogenesis in the striatum of the adult human brain. Cell 156, 1072–1083 24561062 10.1016/j.cell.2014.01.044

[41] Carlén M., Meletis K., Göritz C., Darsalia V., Evergren E., Tanigaki K., Amendola M., Barnabé-Heider F., Yeung M. S., Naldini L., Honjo T., Kokaia Z., Shupliakov O., Cassidy R. M., Lindvall O., and Frisén J. (2009) Forebrain ependymal cells are Notch-dependent and generate neuroblasts and astrocytes after stroke. Nat. Neurosci. 12, 259–267 19234458 10.1038/nn.2268

[42] Luo Y., Coskun V., Liang A., Yu J., Cheng L., Ge W., Shi Z., Zhang K., Li C., Cui Y., Lin H., Luo D., Wang J., Lin C., Dai Z., Zhu H., Zhang J., Liu J., Liu H., deVellis J., Horvath S., Sun Y. E., and Li S. (2015) Single-cell transcriptome analyses reveal signals to activate dormant neural stem cells. Cell 161, 1175–1186 26000486 10.1016/j.cell.2015.04.001 PMC4851109

[43] Tropepe V., Hitoshi S., Sirard C., Mak T. W., Rossant J., and van der Kooy D. (2001) Direct neural fate specification from embryonic stem cells: A primitive mammalian neural stem cell stage acquired through a default mechanism. Neuron 30, 65–78 11343645 10.1016/s0896-6273(01)00263-x

[44] Costa M. R., Kessaris N., Richardson W. D., Götz M., and Hedin-Pereira C. (2007) The marginal zone/layer I as a novel niche for neurogenesis and gliogenesis in developing cerebral cortex. J. Neurosci. 27, 11376–11388 17942732 10.1523/JNEUROSCI.2418-07.2007 PMC6673031

[45] Doetsch F., Petreanu L., Caille I., Garcia-Verdugo J. M., and Alvarez-Buylla A. (2002) EGF converts transit-amplifying neurogenic precursors in the adult brain into multipotent stem cells. Neuron 36, 1021–1034 12495619 10.1016/s0896-6273(02)01133-9

[46] Rietze R. L., Valcanis H., Brooker G. F., Thomas T., Voss A. K., and Bartlett P. F. (2001) Purification of a pluripotent neural stem cell from the adult mouse brain. Nature 412, 736–739 11507641 10.1038/35089085

[47] Kim M., and Morshead C. M. (2003) Distinct populations of forebrain neural stem and progenitor cells can be isolated using side-population analysis. J. Neurosci. 23, 10703–10709 14627655 10.1523/JNEUROSCI.23-33-10703.2003 PMC6740907

[48] Keyoung H. M., Roy N. S., Benraiss A., Louissaint A. Jr., Suzuki A., Hashimoto M., Rashbaum W. K., Okano H., and Goldman S. A. (2001) High-yield selection and extraction of two promoter-defined phenotypes of neural stem cells from the fetal human brain. Nat. Biotechnol. 19, 843–850 11533643 10.1038/nbt0901-843

[49] Nakano I., Paucar A. A., Bajpai R., Dougherty J. D., Zewail A., Kelly T. K., Kim K. J., Ou J., Groszer M., Imura T., Freije W. A., Nelson S. F., Sofroniew M. V., Wu H., Li X., Terskikh A. V., Geschwind D. H., and Kornblum H. I.(2005) Maternal embryonic leucine zipper kinase (MELK) regulates multipotent neural progenitor proliferation. J. Cell Biol. 170, 413–427 16061694 10.1083/jcb.200412115 PMC2171475

[50] Capela A., and Temple S. (2002) LeX/ssea-1 is expressed by adult mouse CNS stem cells, identifying them as nonependymal. Neuron 35, 865–875 12372282 10.1016/s0896-6273(02)00835-8

[51] Uchida N., Buck D. W., He D., Reitsma M. J., Masek M., Phan T. V., Tsukamoto A. S., Gage F. H., and Weissman I. L. (2000) Direct isolation of human central nervous system stem cells. Proc. Natl. Acad. Sci. U.S.A. 97, 14720–14725 11121071 10.1073/pnas.97.26.14720 PMC18985

[52] Nam H.-S., and Benezra R. (2009) High levels of Id1 expression define B1 type adult neural stem cells. Cell Stem Cell 5, 515–526 19896442 10.1016/j.stem.2009.08.017 PMC2775820

[53] Mich J. K., Signer R. A., Nakada D., Pineda A., Burgess R. J., Vue T. Y., Johnson J. E., and Morrison S. J. (2014) Prospective identification of functionally distinct stem cells and neurosphere-initiating cells in adult mouse forebrain eLife 3, e02669 24843006 10.7554/eLife.02669 PMC4038845

[54] Ciccolini F., and Svendsen C. N. (1998) Fibroblast growth factor 2 (FGF-2) promotes acquisition of epidermal growth factor (EGF) responsiveness in mouse striatal precursor cells: Identification of neural precursors responding to both EGF and FGF-2. J. Neurosci. 18, 7869–7880 9742155 10.1523/JNEUROSCI.18-19-07869.1998 PMC6792996

[55] Suzuki M., Wright L. S., Marwah P., Lardy H. A., and Svendsen C. N. (2004) Mitotic and neurogenic effects of dehydroepiandrosterone (DHEA) on human neural stem cell cultures derived from the fetal cortex. Proc. Natl. Acad. Sci. U.S.A. 101, 3202–3207 14973190 10.1073/pnas.0307325101 PMC365767

[56] Geschwind D. H., Ou J., Easterday M. C., Dougherty J. D., Jackson R. L., Chen Z., Antoine H., Terskikh A., Weissman I. L., Nelson S. F., and Kornblum H. I. (2001) A genetic analysis of neural progenitor differentiation. Neuron 29, 325–339 11239426 10.1016/s0896-6273(01)00209-4

[57] Shoemaker L. D., Orozco N. M., Geschwind D. H., Whitelegge J. P., Faull K. F., and Kornblum H. I. (2010) Identification of differentially expressed proteins in murine embryonic and postnatal cortical neural progenitors. PLoS ONE 5, e9121 20161753 10.1371/journal.pone.0009121 PMC2817745

[58] Schulz T. C., Palmarini G. M., Noggle S. A., Weiler D. A., Miltalipova M. M., and Condie B. G. (2003) Directed neuronal differentiation of human embryonic stem cells. BMC Neuroscience 4, 27 14572319 10.1186/1471-2202-4-27 PMC272931

[59] Elkabetz Y., and Studer L. (2008) Human ESC-derived neural rosettes and neural stem cell progression. Cold Spring Harb. Symp. Quant. Biol. 73, 377–387 19204067 10.1101/sqb.2008.73.052

[60] Li X.-J., Du Z.-W., Zarnowska E. D., Pankratz M., Hansen L. O., Pearce R. A., and Zhang S.-C. (2005) Specification of motoneurons from human embryonic stem cells. Nat. Biotechnol. 23, 215–221 15685164 10.1038/nbt1063

[61] Espuny-Camacho I., Michelsen K. A., Gall D., Linaro D., Hasche A., Bonnefont J., Bali C., Orduz D., Bilheu A., Herpoel A., Lambert N., Gaspard N., Péron S., Schiffmann S. N., Giugliano M., Gaillard A., and Vanderhaeghen P. (2013) Pyramidal neurons derived from human pluripotent stem cells integrate efficiently into mouse brain circuits in vivo. Neuron 77, 440–456 23395372 10.1016/j.neuron.2012.12.011

[62] van de Leemput J., Boles N. C., Kiehl T. R., Corneo B., Lederman P., Menon V., Lee C., Martinez R. A., Levi B. P., Thompson C. L., Yao S., Kaykas A., Temple S., and Fasano C. A. (2014) CORTECON: A temporal transcriptome analysis of in vitro human cerebral cortex development from human embryonic stem cells. Neuron 83, 51–68 24991954 10.1016/j.neuron.2014.05.013

[63] Stein J. L., de la, Torre-Ubieta L., Tian Y., Parikshak N. N., Hernández I. A., Marchetto M. C., Baker D. K., Lu D., Hinman C. R., Lowe J. K., Wexler E. M., Muotri A. R., Gage F. H., Kosik K. S., and Geschwind D. H. (2014) A quantitative framework to evaluate modeling of cortical development by neural stem cells. Neuron 83, 69–86 24991955 10.1016/j.neuron.2014.05.035 PMC4277209

[64] Schwanhäusser B., Busse D., Li N., Dittmar G., Schuchhardt J., Wolf J., Chen W., and Selbach M. (2011) Global quantification of mammalian gene expression control. Nature 473, 337–342 21593866 10.1038/nature10098

[65] Reiland S., Salekdeh G. H. and Krijgsveld J. (2011) Defining pluripotent stem cells through quantitative proteomic analysis. Expert Rev. Proteomics 8, 29–42 21329426 10.1586/epr.10.100

[66] Skalnikova H., Vodicka P., Gadher S. J., and Kovarova H. (2008) Proteomics of neural stem cells. Expert Rev. Proteomics 5, 175–186 18466050 10.1586/14789450.5.2.175

[67] Melo-Braga M. N., Meyer M., Zeng X., and Larsen M. R. (2015) Characterization of human neural differentiation from pluripotent stem cells using proteomics/PTMomics—Current state-of-the-art and challenges. Proteomics 15, 656–674 25418965 10.1002/pmic.201400388

[68] Maurer M. H., Feldmann R. E. Jr., Fütterer C. D., and Kuschinsky W. (2003) The proteome of neural stem cells from adult rat hippocampus. Proteome Sci. 1, 4 12818002 10.1186/1477-5956-1-4 PMC165415

[69] Hoffrogge R., Mikkat S., Scharf C., Beyer S., Christoph H., Pahnke J., Mix E., Berth M., Uhrmacher A., Zubrzycki I. Z., Miljan E., Völker U., and Rolfs A. (2006) 2-DE proteome analysis of a proliferating and differentiating human neuronal stem cell line (ReNcell VM). Proteomics 6, 1833–1847 16475233 10.1002/pmic.200500556

[70] Wilhelm M., Schlegl J., Hahne H., Moghaddas Gholami A., Lieberenz M., Savitski M. M., Ziegler E., Butzmann L., Gessulat S., Marx H., Mathieson T., Lemeer S., Schnatbaum K., Reimer U., Wenschuh H., Mollenhauer M., Slotta-Huspenina J., Boese J. H., Bantscheff M., Gerstmair A., Faerber F., and Kuster B. (2014) Mass-spectrometry-based draft of the human proteome. Nature 509, 582–587 24870543 10.1038/nature13319

[71] Angel T. E., Aryal U. K., Hengel S. M., Baker E. S., Kelly R. T., Robinson E. W., and Smith R. D. (2012) Mass spectrometry-based proteomics: Existing capabilities and future directions. Chem. Soc. Rev. 41, 3912–3928 22498958 10.1039/c2cs15331a PMC3375054

[72] Altelaar A. F., Munoz J. and Heck A. J. (2013) Next-generation proteomics: Towards an integrative view of proteome dynamics. Nat. Rev. Genet. 14, 35–48 23207911 10.1038/nrg3356

[73] Zhao Y., and Jensen O. N. (2009) Modification-specific proteomics: Strategies for characterization of post-translational modifications using enrichment techniques. Proteomics 9, 4632–4641 19743430 10.1002/pmic.200900398 PMC2892724

[74] Hudler P., Videtič Paska A. and Komel R. (2015) Contemporary proteomic strategies for clinical epigenetic research and potential impact for the clinic. Expert Rev. Proteomics 12, 197–212 25719543 10.1586/14789450.2015.1019479

[75] Brill L. M., Xiong W., Lee K. B., Ficarro S. B., Crain A., Xu Y., Terskikh A., Snyder E. Y., and Ding S. (2009) Phosphoproteomic analysis of human embryonic stem cells. Cell Stem Cell 5, 204–213 19664994 10.1016/j.stem.2009.06.002 PMC2726933

[76] Buckley S. M., Aranda-Orgilles B., Strikoudis A., Apostolou E., Loizou E., Moran-Crusio K., Farnsworth C. L., Koller A. A., Dasgupta R., Silva J. C., Stadtfeld M., Hochedlinger K., Chen E. I., and Aifantis I. (2012) Regulation of pluripotency and cellular reprogramming by the ubiquitin-proteasome system. Cell Stem Cell 11, 783–798 23103054 10.1016/j.stem.2012.09.011 PMC3549668

[77] Melo-Braga M. N., Schulz M., Liu Q., Swistowski A., Palmisano G., Engholm-Keller K., Jakobsen L., Zeng X., and Larsen M. R. (2014) Comprehensive quantitative comparison of the membrane proteome, phosphoproteome, and sialiome of human embryonic and neural stem cells. Mol. Cell. Proteomics 13, 311–328 24173317 10.1074/mcp.M112.026898 PMC3879623

[78] Venne A. S., Kollipara L., and Zahedi R. P. (2014) The next level of complexity: Crosstalk of posttranslational modifications. Proteomics 14, 513–524 24339426 10.1002/pmic.201300344

[79] Jung H. R., Sidoli S., Haldbo S., Sprenger R. R, Schwämmle V., Pasini D., Helin K., and Jensen O. N. (2013) Precision mapping of coexisting modifications in histone H3 tails from embryonic stem cells by ETD-MS/MS. Anal. Chem. 85, 8232–8239 23889513 10.1021/ac401299w

[80] Prokhorova T. A., Rigbolt K. T., Johansen P. T., Henningsen J., Kratchmarova I., Kassem M., and Blagoev B. (2009) Stable isotope labeling by amino acids in cell culture (SILAC) and quantitative comparison of the membrane proteomes of self-renewing and differentiating human embryonic stem cells. Mol. Cell. Proteomics 8, 959–970 19151416 10.1074/mcp.M800287-MCP200 PMC2689770

[81] Toma J. S., Shettar B. C., Chipman P. H., Pinto D. M., Borowska J. P., Ichida J. K., Fawcett J. P., Zhang Y., Eggan K., and Rafuse V. F. (2015) Motoneurons derived from induced pluripotent stem cells develop mature phenotypes typical of endogenous spinal motoneurons. J. Neurosci. 35, 1291–1306 25609642 10.1523/JNEUROSCI.2126-14.2015 PMC4402330

[82] Severino V., Farina A., Colucci-D'Amato L., Reccia M. G., Volpicelli F., Parente A., and Chambery A. (2013) Secretome profiling of differentiated neural mes-c-myc A1 cell line endowed with stem cell properties. Biochim. Biophys. Acta 1834, 2385–2395 23246712 10.1016/j.bbapap.2012.12.005

[83] Colucci-D'Amato L., Cicatiello A. E., Reccia M. G., Volpicelli F., Severino V., Russo R., Sandomenico A., Doti N., D'Esposito V., Formisano P., and Chambery A. (2015) A targeted secretome profiling by multiplexed immunoassay revealed that secreted chemokine ligand 2 (MCP-1/CCL2) affects neural differentiation in mesencephalic neural progenitor cells. Proteomics 15, 714–724 25404527 10.1002/pmic.201400360

[84] Kim W. K., Kim D., Cui J., Jang H. H., Kim K. S., Lee H. J., Kim S. U., and Ahn S. M. (2014) Secretome analysis of human oligodendrocytes derived from neural stem cells. PLoS ONE 9, e84292 24392122 10.1371/journal.pone.0084292 PMC3879300

[85] Farina A., D'Aniello C., Severino V., Hochstrasser D. F., Parente A., Minchiotti G, and Chambery A. (2011) Temporal proteomic profiling of embryonic stem cell secretome during cardiac and neural differentiation. Proteomics 11, 3972–3982 21770033 10.1002/pmic.201100063

[86] Skalnikova H., Motlik J., Gadher S. J., and Kovarova H. (2011) Mapping of the secretome of primary isolates of mammalian cells, stem cells and derived cell lines. Proteomics 11, 691–708 21241017 10.1002/pmic.201000402

[87] Svetlov S. I., Ignatova T. N., Wang K. K., Hayes R. L., English D., and Kukekov V. G. (2004) Lysophosphatidic acid induces clonal generation of mouse neurospheres via proliferation of Sca-1- and AC133-positive neural progenitors Stem Cells Dev. 13, 685–693 15684836 10.1089/scd.2004.13.685

[88] Dottori M., Leung J., Turnley A. M., and Pébay A. (2008) Lysophosphatidic acid inhibits neuronal differentiation of neural stem/progenitor cells derived from human embryonic stem cells. Stem Cells 26, 1146–1154 18308941 10.1634/stemcells.2007-1118

[89] Li J., Cui Z., Zhao S., and Sidman R. L. (2007) Unique glycerophospholipid signature in retinal stem cells correlates with enzymatic functions of diverse long-chain acyl-CoA synthetases. Stem Cells 25, 2864–2873 17690180 10.1634/stemcells.2007-0308

[90] Almeida R., Pauling J. K., Sokol E., Hannibal-Bach H. K., and Ejsing C. S. (2015) Comprehensive lipidome analysis by shotgun lipidomics on a hybrid quadrupole-Orbitrap-linear ion trap mass spectrometer. J. Am. Soc. Mass Spectrom. 26, 133–148 25391725 10.1007/s13361-014-1013-x

[91] Hansson J., and Krijgsveld J.(2013) Proteomic analysis of cell fate decision. Curr. Opin. Genet. Dev. 23, 540–547 23942315 10.1016/j.gde.2013.06.004

[92] Chaerkady R., Kerr C. L., Marimuthu A., Kelkar D. S., Kashyap M. K., Gucek M., Gearhart J. D., and Pandey A. (2009) Temporal analysis of neural differentiation using quantitative proteomics. J. Proteome Res. 8, 1315–1326 19173612 10.1021/pr8006667 PMC2693473

[93] Chaerkady R., Letzen B., Renuse S., Sahasrabuddhe N. A., Kumar P., All A. H., Thakor N. V., Delanghe B., Gearhart J. D., Pandey A., and Kerr C. L. (2011) Quantitative temporal proteomic analysis of human embryonic stem cell differentiation into oligodendrocyte progenitor cells. Proteomics 11, 4007–4020 21770034 10.1002/pmic.201100107 PMC3728712

[94] Cao R., Chen K., Song Q., Zang Y., Li J., Wang X., Chen P., and Liang S. (2012) Quantitative proteomic analysis of membrane proteins involved in astroglial differentiation of neural stem cells by SILAC labeling coupled with LC–MS/MS. J. Proteome Res. 11, 829–838 22149100 10.1021/pr200677z

[95] Yocum A. K., Gratsch T. E., Leff N., Strahler J. R., Hunter C. L., Walker A. K., Michailidis G., Omenn G. S., O'Shea K. S., and Andrews P. C. (2008) Coupled global and targeted proteomics of human embryonic stem cells during induced differentiation. Mol. Cell. Proteomics 7, 750–767 18304949 10.1074/mcp.M700399-MCP200 PMC2401335

[96] Wei T., Peng X., Ye L., Wang J., Song F., Bai Z., Han G., Ji F., and Lei H (2015) Web resources for stem cell research. Genomics Proteomics Bioinformatics 13, 40–45 25701763 10.1016/j.gpb.2015.01.001 PMC4411488

[97] Perez-Riverol Y., Alpi E., Wang R., Hermjakob H., and Vizcaíno J. A. (2015) Making proteomics data accessible and reusable: Current state of proteomics databases and repositories. Proteomics 15, 930–949 25158685 10.1002/pmic.201400302 PMC4409848

[98] Hussein S. M., Puri M. C., Tonge P. D., Benevento M., Corso A. J., Clancy J. L., Mosbergen R., Li M., Lee D. S., Cloonan N., Wood D. L., Munoz J., Middleton R., Korn O., Patel H. R., White C. A., Shin J. Y., Gauthier M. E., Lê Cao K. A., Kim J. I., Mar J. C., Shakiba N., Ritchie W., Rasko J. E., Grimmond S. M., Zandstra P. W., Wells C. A., Preiss T., Seo J. S., Heck A. J., Rogers I. M., and Nagy A. (2014) Genome-wide characterization of the routes to pluripotency. Nature 516, 198–206 25503233 10.1038/nature14046

[99] Wells C. A., Mosbergen R., Korn O., Choi J., Seidenman N., Matigian N. A., Vitale A. M., and Shepherd J. (2013) Stemformatics: Visualisation and sharing of stem cell gene expression. Stem Cell Res. 10, 387–395 23466562 10.1016/j.scr.2012.12.003

[100] Bandura D. R., Baranov V. I., Ornatsky O. I., Antonov A., Kinach R., Lou X., Pavlov S., Vorobiev S., Dick J. E., and Tanner S. D. (2009) Mass cytometry: Technique for real time single cell multitarget immunoassay based on inductively coupled plasma time-of-flight mass spectrometry. Anal. Chem. 81, 6813–6822 19601617 10.1021/ac901049w

[101] Zunder E. R., Lujan E., Goltsev Y., Wernig M., and Nolan G. P. (2015) A continuous molecular roadmap to iPSC reprogramming through progression analysis of single-cell mass cytometry. Cell Stem Cell 16, 323–337 25748935 10.1016/j.stem.2015.01.015 PMC4401090

[102] Stoevesandt O., and Taussig M. J. (2012) Affinity proteomics: The role of specific binding reagents in human proteome analysis. Expert Rev. Proteomics 9, 401–414 22967077 10.1586/epr.12.34

[103] Uhlen M., Oksvold P., Fagerberg L., Lundberg E., Jonasson K., Forsberg M., Zwahlen M., Kampf C., Wester K., Hober S., Wernerus H., Björling L., and Ponten F. (2010) Towards a knowledge-based Human Protein Atlas. Nat. Biotechnol. 28, 1248–1250 21139605 10.1038/nbt1210-1248

[104] Byström S., Ayoglu B., Häggmark A., Mitsios N., Hong M. G., Drobin K., Forsström B., Fredolini C,. Khademi M., Amor S., Uhlén M., Olsson T., Mulder J., Nilsson P., and Schwenk J. M. (2014) Affinity proteomic profiling of plasma, cerebrospinal fluid, and brain tissue within multiple sclerosis. J. Proteome Res. 13, 4607–4619 25231264 10.1021/pr500609e

[105] Chu C., Zhang Q. C., da Rocha S. T., Flynn R. A., Bharadwaj M., Calabrese J. M., Magnuson T., Heard E., and Chang H. Y. (2015) Systematic discovery of Xist RNA binding proteins. Cell 161, 404–416 25843628 10.1016/j.cell.2015.03.025 PMC4425988

[106] Ramos A. D., Diaz A., Nellore A., Delgado R. N., Park K. Y., Gonzales-Roybal G., Oldham M. C., Song J. S., and Lim D. A. (2013) Integration of genome-wide approaches identifies lncRNAs of adult neural stem cells and their progeny in vivo. Cell Stem Cell 12, 616–628 23583100 10.1016/j.stem.2013.03.003 PMC3662805

[107] Mercer T. R., Qureshi I. A., Gokhan S., Dinger M. E., Li G., Mattick J. S., and Mehler M. F. (2010) Long noncoding RNAs in neuronal-glial fate specification and oligodendrocyte lineage maturation. BMC Neuroscience 11, 14 20137068 10.1186/1471-2202-11-14 PMC2829031

[108] Engelen E., Brandsma J. H., Moen M. J., Signorile L., Dekkers D. H. W., Demmers J., Kockx C. E. M., Ozgur Z., van Ijcken W. F. J., Van den Berg D. L. C., and Poot R. A. (2015) Proteins that bind regulatory regions identified by histone modification chromatin immunoprecipitations and mass spectrometry. Nat. Commun. 6, 1–12 10.1038/ncomms8155 PMC4455091 25990348

[109] Eckfeldt C. E., Mendenhall E. M., and Verfaillie C. M. (2005) The molecular repertoire of the ‘almighty’ stem cell. Nat. Rev. Mol. Cell Biol. 6, 726–37 16103873 10.1038/nrm1713

[110] Van Hoof D., Heck A. J. R., Krijgsveld J., and Mummery C. L. (2008) Proteomics and human embryonic stem cells. Stem Cell Res. 1, 169–182 19383398 10.1016/j.scr.2008.05.003

